# Metastatic triple-negative breast cancer – current treatment strategies in the Nordics: a modified Delphi study

**DOI:** 10.2340/1651-226X.2025.42733

**Published:** 2025-03-05

**Authors:** Antonios Valachis, Peeter Karihtala, Jürgen Geisler, Malgorzata K. Tuxen

**Affiliations:** a Department of Oncology, Faculty of Medicine and Health, Örebro University, Örebro, Sweden; b Department of Oncology, Helsinki University Hospital Comprehensive Cancer Centre and University of Helsinki, Helsinki, Finland; c Department of Oncology, Division of Medicine, Akershus University Hospital (AHUS), Lørenskog, Norway; d Institute for Clinical Medicine, Faculty of Medicine, University of Oslo, Campus AHUS, Oslo, Norway; e Department of Oncology, Herlev and Gentofte University Hospital, Herlev, Denmark

**Keywords:** mTNBC, Delphi method, Treatment guidelines, Nordics

## Abstract

**Background and purpose:**

This study aimed to assess current treatment strategies for metastatic triple-negative breast cancer (mTNBC) and the perceptions of clinical experts in Sweden, Denmark, Norway, Finland, and Iceland, comparing them to international guidelines to provide insights into how these therapies are implemented and adapted to national Nordic guidelines.

**Methods:**

A three-round modified Delphi method was followed with consensus defined as 70% agreement. A steering committee selected 20 experienced oncologists as panellists and developed the questionnaires. Questions included items related to treatment preferences in different treatment lines with different clinical scenarios in mTNBC patients.

**Results:**

In the first round, eight out of 33 questions on clinical treatment reached consensus with 14 out of 27 in the second round reaching consensus. In round three, eight out of eight questions reached consensus. The preferred treatment for mTNBC patients with PD-L1 positive was checkpoint inhibitors (CPI) in combination with chemotherapy. For patients with germline *BRCA* mutation and PD-L1 negative disease, PARP-inhibitors were preferred as 1L and sacituzumab govitecan (SG) in both 2L and later lines. Disagreement was observed for chemotherapy in later lines where evidence is sparse or lacking.

**Interpretation:**

The high level of consensus for new treatment strategies, such as CPI and PARP-inhibitors in 1L and SG in 2L or later lines, in comparison with the limited consensus for older treatments, such as chemotherapy, may reflect the growing academic evidence for different treatment strategies. Understanding the treatment patterns across different countries contributes to gaining consensus on the upcoming therapeutic advances.

## Introduction

Breast cancer is the second most commonly diagnosed cancer in the Nordics accounting for 13.2% of all cancer types, in both sexes in 2022 [1]. Despite the increase in survival rates [2], breast cancer is still the third most common cause of cancer-related death in women in the Nordics [1].

Triple-negative breast cancer (TNBC) is characterized by the lack of estrogen and progesterone hormone receptors (HR-negative) and lower or missing expression of human epidermal growth factor receptor 2 (HER2-negative [HER2-zero or HER2-low]) [3], which makes it a biologically heterogeneous cancer that is challenging to treat due to the limited access to effective treatment options [4].

During the last 5 years, novel treatment strategies against metastatic TNBC (mTNBC) have emerged, showing survival benefits in various treatment lines compared to traditional therapies. Immune checkpoint inhibitors (CPI) combined with chemotherapy have been established as the preferred first-line (1L) treatment in patients with mTNBC and expression of PD-L1 due to a survival benefit compared to chemotherapy alone [5]. Lately, antibody–drug conjugates (ADCs) such as sacituzumab govitecan (SG) or trastuzumab–deruxtecan (T-DXd; only for HER2-low disease) have been approved due to documented progression-free benefit compared to traditional chemotherapy [6–8]. Another emerging approach to treat mTNBC patients includes the use of PARP-inhibitors. Approximately 10–20% of mTNBC patients carry germline *BRCA1/2* mutations [9] and in this patient subgroup treatment with olaparib or talazoparib showed improved PFS compared to standard chemotherapy [10, 11].

Although treatment guidelines for mTNBC have been developed, for example, by the European Society for Medical Oncology (ESMO) [12], the emerging treatment landscape for patients with mTNBC poses challenges to oncologists, considering the limited evidence on treatment sequencing and the effectiveness of new treatment strategies in specific clinical situations. Thus, this study aimed to assess the perceptions of clinical experts in the Nordics: Sweden, Denmark, Norway, Finland, and Iceland – on current treatment strategies for mTNBC using a modified Delphi method to provide insights into how these therapies are implemented based on current scientific evidence.

## Methods

### Study design and expert panellists

A three-round modified Delphi method (Supplementary Figure 1) was used in the study, which was performed between July 2023 and January 2024. The Delphi method follows a systematic and structured approach to collect expert opinions aiming to reach a consensus on specific topics, and it is used in different medical fields, including oncology [13]. A steering committee (SC), formed by four oncologists with expertise in breast cancer, developed the questionnaires and selected 20 experienced oncologists as panellists, thus, totaling 24 oncology experts in the study. The selection criteria were based on choosing oncologists with experience in treating breast cancer patients and representing different districts of the eligible countries. The questionnaires, included both open- and closed-ended questions, and were in English to ensure anonymity. Panellists were informed to vote based on scientific evidence and, for some specific questions based on treatment availability. Rounds 1 and 2 were conducted in virtual meetings, and round 3 in a hybrid form.

### Round 1

In round 1, 53 questions were included and covered general background information of the experts (20 questions), treatment choices in different lines and clinical situations, and questions independent of the treatment line (33 questions), similar to a previous Delphi study published by the same SC about the treatment landscape of HR-positive/HER2-negative advanced breast cancer in the Nordics [14]. The questionnaire was shared with all experts, describing the study, aim, and instructions. This round aimed at exploring the treatment strategies used by clinical experts for the treatment of mTNBC, as well as their perceptions. The questionnaire included questions related to both daily clinical practice and current scientific evidence (Supplementary Table 4).

### Round 2

The second round included 28 questions and aimed at reaching a consensus on topics where disagreement was observed in round 1 (Supplementary Table 4). In addition, five cases based on real patient scenarios were included, presenting different clinical scenarios with questions about treatments in different lines (see section Patient cases in Methods).

### Round 3

The questionnaire for round 3 included seven questions covering topics that did not reach consensus in previous rounds. Moreover, two new fictional patient cases were included (Supplementary Table 4). The hybrid meeting was attended by 19 experts on-site and three experts virtually. Two experts were not available for the meeting, of whom one expert was available to vote within 1 month. The hybrid nature of this round allowed discussions between experts about topics that did not reach consensus in a non-anonymous manner.

### Patient cases

Six of the seven patient cases included in the study are presented in this publication. The remaining patient case, which was not related to mTNBC but to post-neoadjuvant setting, will be presented elsewhere. The treatment given to patients in the cases was prespecified by the SC and it was used to give context for the consensus-seeking questions related to different treatment lines.

### Data analysis and interpretation

The data collected in each questionnaire were anonymous to the other panellists. Questionnaires of rounds 1 and 2 were shared with the experts via SurveyMonkey^®^ [15]. Round 3 was performed using the web-based polling system Pigeonhole Live™ [16]. After each round, the SC held a meeting to analyze the results and prepare the following questionnaire covering topics that did not reach a consensus.

The consensus level for Delphi studies is not universally agreed upon and varies in the literature from 50 to 97% [13]. In this study, considering the number of experts, the aim of the research and the resources, it was defined at a 70% agreement level, while 50–<70% of the agreement was considered as somewhat consensus, and <50% was considered as consensus was not reached. Importantly, experts voted based on current scientific evidence. Participation in clinical trials was not included as a treatment option.

## Results

### Steering committee and panellists

A total of 24 Nordic oncology experts participated in the study, with 20 selected as panellists and four as members of the SC. Most (70.8%) practiced at a university hospital level. About half (45.8%) had over 20 years, 29.2% had 11–20 years, and 25.0% had 6–10 years of clinical experience in treating breast cancer, and 62.5% were National coordinating investigators in clinical trials. Most (83.3%) attended 2–5 international breast cancer conferences annually. Detailed expert characteristics are provided in Supplementary Table 1.

### Outcomes

In the first round, eight out of 33 (24.2%) questions on clinical treatment reached consensus. In the second round, 14 out of 27 (51.9%) questions reached consensus, and in round three, nine out of 12 (75.0%) questions did. Results related to general clinical questions are presented in Supplementary Table 2, and results for questions voted based on clinical practice and reimbursement status are presented in Supplementary Table 3.

### First line treatment

Consensus was reached for treatment choice 1L for patients with mTNBC, PD-L1 positive, no germline *BRCA* mutation, and with disease recurrence at least 3 years after the end of adjuvant standard taxane- and anthracycline-based chemotherapy (from here onwards referred to as after end of adjuvant chemotherapy), who have not received CPI treatment in neither neoadjuvant nor adjuvant setting and have not received post-neoadjuvant therapy. In this case, consensus was unanimously reached to choose CPI plus chemotherapy (Table 1). If the options included only chemotherapy, no consensus was reached (Table 1).

**Table 1:** Main themes of the study.

Theme	Question	Consensus	Consensus statement
1L therapy	Preferred treatment choice in case of PD-L1+ and recurrence after 3 years	100%	CPI + chemotherapy
	Treatment choice/Preferred if PD-L1+ and recurrence after 3 years if chemotherapy is chosen as monotherapy	NR	-
	Treatment choice for backbone chemotherapy if pembrolizumab with chemotherapy is chosen	NR	-
	Treatment choice for backbone chemotherapy if atezolizumab with chemotherapy is chosen	87.5%	Nab-paclitaxel
	Treatment choice for TNBC, PD-L1– with germline *BRCA* mutation and recurrence 3 years after end of chemotherapy	82.6%	PARP-inhibitors
	Treatment choice for TNBC, PD-L1– with germline *BRCA* mutation and recurrence within 1 year after end of chemotherapy	73.9%	PARP-inhibitors
	Treatment choice for PD-L1+ and recurrence within 1 year	83.3%	Pembrolizumab + chemotherapy
	Treatment choice for PD-L1– and recurrence over 3 years	NR	-
	Treatment choice for PD-L1– and recurrence within 1 year	NR	-
	Time period from the end of adjuvant pembrolizumab until disease recurrence before considering initiating 1L treatment with CPI plus chemotherapy	58.3%	More than 12 months
2L therapy	Treatment choice for mTNBC and no germline *BRCA* mutation	62.5%	Sacituzumab govitecan as monotherapy
3L therapy	Treatment choice for mTNBC and no germline *BRCA* mutation	66.7%	Sacituzumab govitecan as monotherapy

BRCA: breast cancer gene; CPI: checkpoint inhibitors; mTNBC: metastatic triple negative breast cancer; NR: not reached; PD-L1: Programmed Cell Death Ligand 1.

In this same clinical situation, if pembrolizumab was used in 1L, no agreement was reached for backbone chemotherapy, but if atezolizumab was used, agreement was reached to use nab-paclitaxel (87.5%) (Table 1). If the options included only chemotherapy, somewhat of a consensus was reached for 1L treatment to choose either carboplatin-based chemotherapy (62.5%), capecitabine as monotherapy (62.5%), or eribulin as monotherapy (62.5%).

Significant agreement was observed to use PARP-inhibitors in 1L for patients with mTNBC, PD-L1 negative and germline *BRCA* mutation who had disease recurrence both at least 3 years after the end of adjuvant chemotherapy (82.6%) or within less than 1 year (73.9%) (Table 1).

Agreement was reached to use pembrolizumab with chemotherapy (83.3%) as 1L treatment for PD-L1 positive patients with disease recurrence within 1 year after the end of adjuvant chemotherapy (no exposure to CPI in curative setting). On the contrary, for patients with PD-L1 negative disease no consensus was reached for 1L treatment (Table 1).

Regarding the time from the end of adjuvant pembrolizumab until disease recurrence, before considering initiating 1L treatment with CPI plus chemotherapy, somewhat of a consensus was achieved for more than 12 months (58.3%) (Table 1).

### Second and third line treatment

Voted as a ranking, some consensus was reached to prescribe SG as monotherapy in second line (2L) as first choice (62.5%) and 20.8% as second choice (a total of 83.3%) and in third line (3L) (66.7%) for patients with no germline *BRCA* mutation (Table 1). These scenarios were further explored in the patient cases 2 and 6.

### General clinical questions

The clinical aspect unanimously selected as the reason not to offer later treatment lines in patients with mTNBC was ‘impaired performance status’. In addition, reducing symptoms related to the primary tumors was the statement that reached consensus (91.7%) as a situation where primary tumor removal for a patient could be considered in patients with *de novo* advanced breast cancer. Consensus was also reached for testing for dihydropyrimidine dehydrogenase (DPD) deficiency before treating patients with capecitabine, according to the European Medicines Agency recommendation [17], and for all questions related to patients with newly diagnosed brain metastases (Supplementary Table 2).

### Patient case 1 – Germline BRCA1-mutation and PD-L1 negative with focus on later treatment lines

The patient was a 30-year-old woman with TNBC – locally advanced breast cancer (cT3cN1). She underwent neoadjuvant therapy with epirubicin with cyclophosphamide (EC-90) and taxanes, followed by surgery and classified as ypT0, pN0 (0/3), and receiving radiation. After 5 years of observation, she was diagnosed with liver metastases (TNBC, germline-mutated *BRCA1*, and PD-L1 negative).

**Figure 1:**
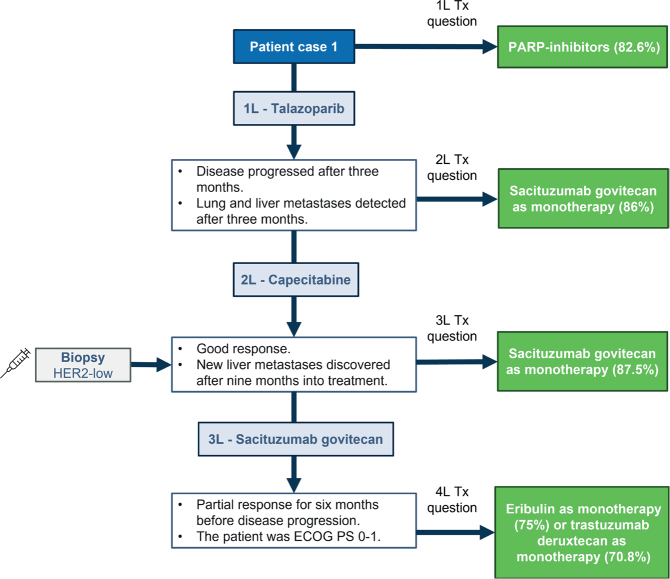
Patient case 1. Treatments on the right side correspond to the choices that experts voted for, that is, the hypothetical treatments for the patient. ECOG PS: Eastern Cooperative Oncology Group performance status; HER2: human epidermal growth factor receptor 2; IHC: immunohistochemistry; PARP: poly-ADP ribose polymerase; Tx: treatment.

Consensus was reached on using PARP-inhibitors in the 1L setting (82.6%). The patient received talazoparib in 1L but experienced disease progression. SG in monotherapy was voted as the 2L (86%) treatment whereas the patient in the case received capecitabine as the 2L treatment. Consensus was also reached to use SG as monotherapy in 3L (87.5%). After disease progression, consensus was reached for treatment in 4L, with either eribulin as monotherapy (75%) or T-DXd as monotherapy (70.8%) (Figure 1).

### Patient case 2 – No BRCA-mutation, PD-L1 positive with focus on later treatment lines

The patient was a 41-year-old woman with TNBC (cT1N0). She underwent breast-conserving surgery and sentinel node dissection. The tumor was classified as pT1bN0. She received adjuvant chemotherapy with three cycles of EC90 followed by three cycles of docetaxel and radiation therapy to the breast. No germline *BRCA* mutation was found. Approximately 10 months after the end of adjuvant chemotherapy, she was diagnosed with liver and lung metastases (TNBC with HER2 IHC 0, PD-L1 positive). The patient received atezolizumab and nab-paclitaxel as 1L therapy. A disease progression was evident after 3 months.

**Figure 2:**
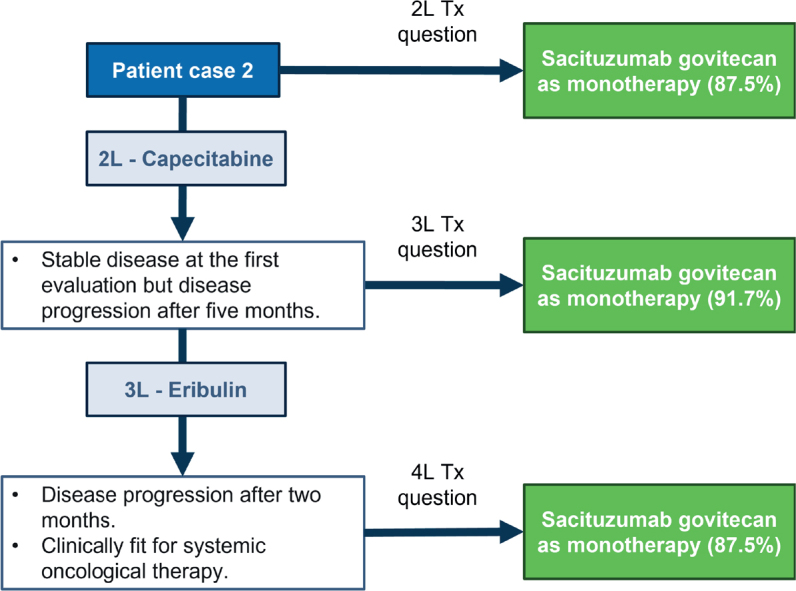
Patient case 2. Treatments on the right side correspond to the choices that experts voted for, that is, the hypothetical treatments for the patient. Tx: treatment.

In this clinical situation, an agreement was reached to use SG as monotherapy in 2L (87.5%). The patient received capecitabine with disease progression after 5 months, SG as monotherapy was also chosen in 3L (91.7%). In case the patient received eribulin in 3L but had a disease progression after 2 months, SG as monotherapy was also chosen as 4L treatment (87.5%) (Figure 2).

### Patient case 3 – De novo mTNBC, BRCA-wt, PD-L1 positive and development of brain metastases

The patient was a 61-year-old woman with TNBC (27 mm estrogen receptor [ER] 5%, progesterone receptor [PR] 0%, HER2 IHC 0 tumor of ductal type, grade 3, Ki67 80%, PAM50 basal-like type, lymph node-negative and *BRCA* negative, clinical N0). She underwent left breast-conserving surgery and a sentinel node showing 2 out of 2 lymph node metastases. Postoperative CT scan showed small lung metastases (PD-L1 positive) originating from breast cancer. She had asthma and hypertension and was ECOG PS 1. One month after surgery, the patient started treatment with atezolizumab plus nab-paclitaxel, but she developed autoimmune hepatitis after six cycles and continued monotherapy with nab-paclitaxel. One month later, she had progression in the lung and had three liver metastases. She continued having ECOG PS 1 and normal liver function.

**Figure 3:**
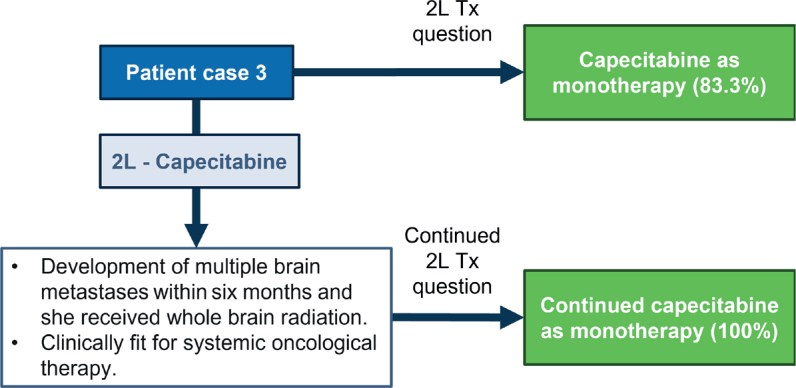
Patient case 3. Treatments on the right side correspond to the choices that experts voted for, that is, the hypothetical treatments for the patient. Tx: treatment.

Based on the patient characteristics, a consensus was reached to use capecitabine as monotherapy in 2L (83.3%) – note that 2L *de novo* patients are not within the SG label. However, the patient developed multiple brain metastases within 6 months. After receiving whole-brain radiation, consensus was reached to continue capecitabine treatment until further progression (100%) (Figure 3).

### Patient case 4 – Germline BRCA1-mutation, PD-L1 negative

The patient was a 38-year-old premenopausal woman with TNBC (26 mm [measured by breast ultrasound] ER negative, PR negative, HER2 IHC 0, invasive ductal carcinoma, grade 3, in the right breast, lymph node-positive, *BRCA1* positive, clinical N0). She received neoadjuvant chemotherapy – three cycles of epirubicin and cyclophosphamide every 3 weeks and three cycles of paclitaxel weekly. She underwent conserving surgery in the right breast and sentinel node that resulted in a pathological partial response (ypT1bN0). She received adjuvant radiation therapy to the breast with a boost due to her young age. After 16 months, she experienced recurrence with bone and liver metastases (TNBC and PD-L1 negative). She received six cycles of carboplatin and gemcitabine and had progression in the liver.

**Figure 4:**
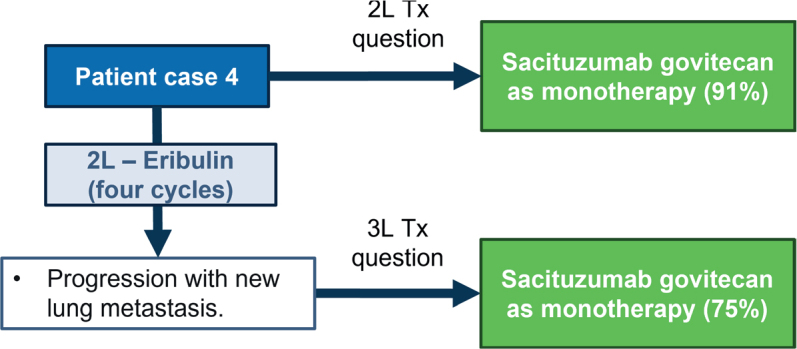
Patient case 4. Treatments on the right side correspond to the choices that experts voted for, that is, the hypothetical treatments for the patient. Tx: treatment.

A consensus was reached to use SG as monotherapy in 2L (91%). In case the patient received eribulin for four cycles in 2L and had progression with new lung metastasis, agreement on using SG as monotherapy in 3L (75%) was reached (Figure 4).

### Patient case 5 –ER-low and PD-L1 positive breast cancer

**Figure 5:**
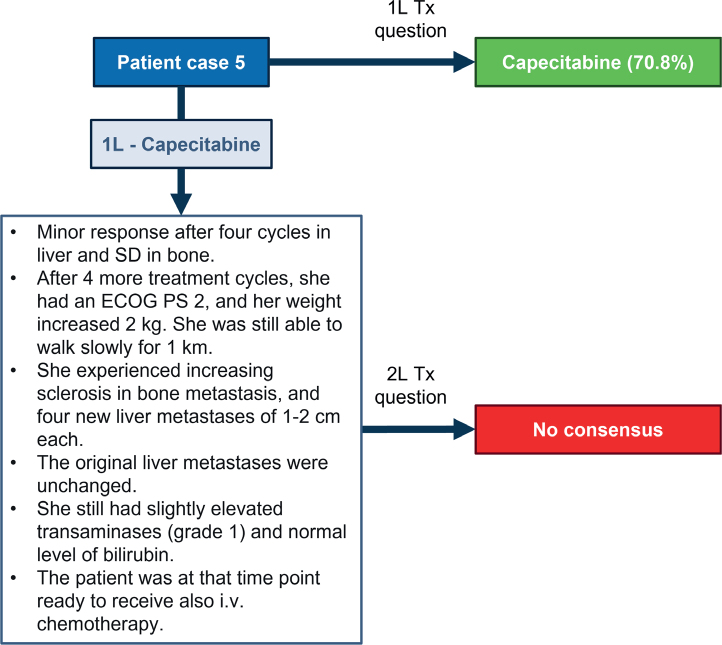
Patient case 5. Treatments on the right side correspond to the choices that experts voted for, that is, the hypothetical treatments for the patient. ECOG PS: Eastern Cooperative Oncology Group performance status; SD: stable disease; Tx: treatment.

The patient was a 68-year-old woman with breast cancer (17 mm grade III invasive ductal carcinoma, ER 15%, PR 0%, HER2 IHC 2+ [but ISH–], Ki-67 35%). She underwent breast-conserving surgery on the right breast and a sentinel node biopsy. She received three cycles of docetaxel plus three of cycles of cyclophosphamide, epirubicin and fluorouracil (CEF). She had symptomatic pneumonitis from docetaxel, and she did not want to continue CEFs due to fatigue. She had radiotherapy for the breast and axilla, which ended 6 months after surgery. She initiated treatment with letrozole. About 4 years after the initial diagnosis, the patient decreased in weight, around 5 kg over a short period, and experienced pain in her right upper stomach. A whole-body CT showed lytic bone metastases in the thoracic vertebrae II-IV (Th II-IV) and the sacrum. She had up to 10 liver metastases (1–3 cm). Biopsy from the largest metastasis revealed ductal carcinoma, ER 5%, PR 0%, HER2 IHC 0, Ki-67 50% and PD-L1 positive. She had a PS ECOG 2, required daily mild opioids, and was able to walk slowly for 1 km. A consensus was reached to use capecitabine as 1L treatment (70.8%). The patient preferred capecitabine as she was afraid of the side effects of intravenous chemotherapy. No consensus was reached for 2L treatment based on the patient characteristics developed after 1L treatment with capecitabine described in Figure 5.

### Patient case 6 – BRCA-wt, PD-L1 positive with focus on later treatment lines

**Figure 6:**
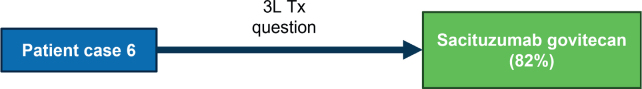
Patient case 6. Treatments on the right side correspond to the choices that experts voted for, that is, the hypothetical treatments for the patient. Tx: treatment.

The patient was a 55-year-old woman with metastatic (lungs and liver) TNBC that recurred 4 years after adjuvant treatment with anthracyclines and taxanes. The disease was ER 0%, PgR 0%, and HER2 IHC 1+. No germline *BRCA* mutation was detected, whereas PD-L1 CPS was 20. The patient received pembrolizumab and paclitaxel in 1L. An initial partial response was noted, but a disease progression was evident 11 months after treatment initiation. She received capecitabine as 2L with stable disease to be the best response and disease progression after 6 months. In this clinical situation, consensus was reached to use SG over T-DXd as 3L (82%) (Figure 6).

## Discussion

This Delphi expert consensus study provides insights into how new treatment strategies are perceived and incorporated into the treatment landscape for patients with mTNBC in the Nordics. It also reflects the challenges oncologists face when emerging treatments are developed, sheds light on treatment approaches in specific clinical scenarios, and considers different treatment aspects in patients with mTNBC. Thus, this study aimed to map the current treatment strategies and perceptions of clinical experts in the Nordics on how patients with mTNBC would be treated based on current scientific evidence and clinical experience.

A unanimous consensus was reached regarding treatment choices in 1L, using combination treatment of CPI with chemotherapy in patients with PD-L1 positive mTNBC, according to approved indications. This result is in line with the ESMO 1L treatment guidelines for mTNBC, which recommend the use of pembrolizumab in combination with chemotherapy (paclitaxel, nab-paclitaxel or carboplatin–gemcitabine) or atezolizumab in combination with nab-paclitaxel [12]. The use of pembrolizumab was preferred over atezolizumab in this setting among the majority of the panellists. A major consensus was achieved to use nab-paclitaxel as backbone chemotherapy when atezolizumab is used in 1L patients, thus reflecting that this combination treatment is adopted in the clinics based on the positive PFS results in patients with mTNBC in the IMpassion130 study [18] and the negative results of IMpassion131 study when atezolizumab was combined with paclitaxel [19]. On the other hand, the results in this Delphi study, in which no consensus was reached for backbone chemotherapy when using pembrolizumab, reflect the different alternatives for backbone treatments that were used in the KEYNOTE-335 trial [5]. In fact, the KEYNOTE-355 trial compared pembrolizumab plus the investigator’s choice of chemotherapy, which included nanoparticle albumin-bound paclitaxel, paclitaxel, or gemcitabine–carboplatin versus placebo plus chemotherapy in patients with previously untreated locally recurrent inoperable or mTNBC and showed OS benefit in favor of combination irrespective of the type of backbone chemotherapy [5].

In patients with germline *BRCA* mutation and PD-L1 negative mTNBC, consensus was achieved in utilizing PARP-inhibitors as a 1L treatment option compared to any chemotherapeutic agent. The question did not differentiate the use of olaparib or talazoparib. These results are in agreement with ESMO guidelines that recommend the use of olaparib or talazoparib in this clinical situation [12] based on the positive results of OlympiAD and EMBRACA clinical trials where PARP-inhibitors resulted in improved PFS compared to standard chemotherapy [10, 11]. Interestingly, neither OlympiAD nor EMBRACA included patients who were treated in a 1L setting. The fact that PARP-inhibitors are suggested as the preferred treatment option in 1L setting for patients with germline *BRCA*-mutated mTNBC is mainly attributed to their targeted mechanism of action and their favorable toxicity profile compared to conventional chemotherapy [10, 11].

Interestingly, no consensus was reached where only chemotherapeutic agents were the available treatment options, except the use of carboplatin-based chemotherapy as 1L treatment if this treatment option has not been used as part of neoadjuvant therapy and the use of eribulin or capecitabine in later lines, as depicted in patient cases 1 and 3, respectively. As few randomized controlled trials investigating different chemotherapeutic agents dedicated to patients with mTNBC have been performed, current clinical practice mostly reflects local traditions and clinical experience thus making the consensus across countries challenging. Notably, the use of platinum-based chemotherapy as 1L, as well as the choice of eribulin in later lines, are supported by randomized evidence [20–22], whereas the convenience in administrating capecitabine together with the long-term clinical experience could explain the consensus for this treatment option.

Regarding questions about treatment choices in 2L, SG reached consensus as the preferred option in both 2L and later treatment lines compared to any chemotherapeutic agent in patients with mTNBC and no *BRCA* mutation (patient case 2). Also, in this clinical situation, results from the study follow the ESMO guidelines in 2L [12], which recommends SG as 2L treatment. The evidence supporting the usage of SG instead of chemotherapy in patients with mTNBC is based on results of the phase 3 ASCENT trial [6, 7]. In addition, in patients with mTNBC and HER2-low disease, consensus was achieved on the preference of using SG instead of T-DXd or chemotherapeutic agents in later treatment lines. These results are exemplified in patient case 1 and 6 and are justified by the certainty of evidence for these ADCs in mTNBC. In fact, the benefit of SG in this treatment setting is supported by the results of a randomized trial dedicated to patients with mTNBC [6, 7] whereas the benefit of T-DXd has been shown in a pre-defined subgroup analysis of a randomized trial [8].

Interestingly, two of the cases in Round 2 described patients with ER-low disease who were treated as triple-negative. The treatment strategy for patients with ER-low disease remains a topic of debate, with a growing body of evidence suggesting that ER-low resembles ER-negative rather than ER-positive disease [23, 24]. In Nordic countries, patients with ER <10% have traditionally been treated as having ER-negative disease. Thus, including patient cases with ER-low disease in the Delphi study could help guide potential treatment approaches for this patient subgroup while awaiting further evidence

The results of the present study should be interpreted in the light of specific methodological strengths and limitations. Applying the Delphi method has several advantages, such as anonymity, flexibility, and enhanced validation of the data collected in different rounds [25]. In addition, including a broad group of expert oncologists with a high level of expertise further strengthened the study. The limitations of this methodology should also be considered, as being highly sensitive to the study design, including the panel of experts’ expertise and composition, and the detail of clarity in the questions [25]. One limitation of including five cases based on real patient scenarios in Round 2 is that some treatment choices may be considered outdated based on current clinical practice. However, using real patient scenarios ensures that the cases reflect the challenges clinicians face in daily practice. Another limitation that should be acknowledged is that some clinical scenarios may not have been captured in any of the rounds. One particularly challenging situation is the choice of first-line treatment for patients with gBRCA-mutated, PD-L1-positive metastatic disease, where both CPIs and PARP inhibitors are viable options. Since gBRCA mutations do not appear to impact CPI effectiveness in either the early or metastatic setting [26, 27], and delaying CPI use to later lines when disease has been progressed to chemotherapy may reduce its benefit [28], one could argue that CPI should be the preferred first-line option for this group, while PARP inhibitors remain a valid choice for later lines.

In summary, a high level of consensus for new treatment strategies, such as CPI and PARP-inhibitors in 1L and SG in 2L or later lines, was observed compared with the limited consensus for older treatment options, such as chemotherapy. These results may reflect the strong evidence existing for the different treatment strategies. An important prerequisite observed in the study was that to achieve any consensus, the focus should be on scientific evidence rather than clinical practice since reimbursement statuses can differ across Nordic countries. Finally, as the treatment landscape for mTNBC emerges with more robust scientific evidence on treatment sequencing and novel treatments, similar efforts to understand the treatment patterns across countries should be intensified to gain consensus on the upcoming therapeutic advances.

## Supplementary Material

Metastatic triple-negative breast cancer – current treatment strategies in the Nordics: a modified Delphi study

## Data Availability

The data supporting this study’s findings are available on request from the corresponding author (A.V.). However, the data are not publicly available due to restrictions, such as their containing information that could compromise the privacy of research participants.
