# Supplementary material for: Metastatic triple-negative breast cancer – current treatment strategies in the Nordics: a modified Delphi study
Source: Acta Oncol. 2025 Mar 5;64:42733. doi: 10.2340/1651-226X.2025.42733 (PMC11898304; doi:10.2340/1651-226X.2025.42733)
Supplement: Metastatic triple-negative breast cancer – current treatment strategies in the Nordics: a modified Delphi study [file AO-64-42733-s1.pdf]

Supplementary material has been published as submitted. It has not been copyedited, or typeset by Acta Oncologica

Supplementary Figure 1. Modified TNBC Delphi study process.

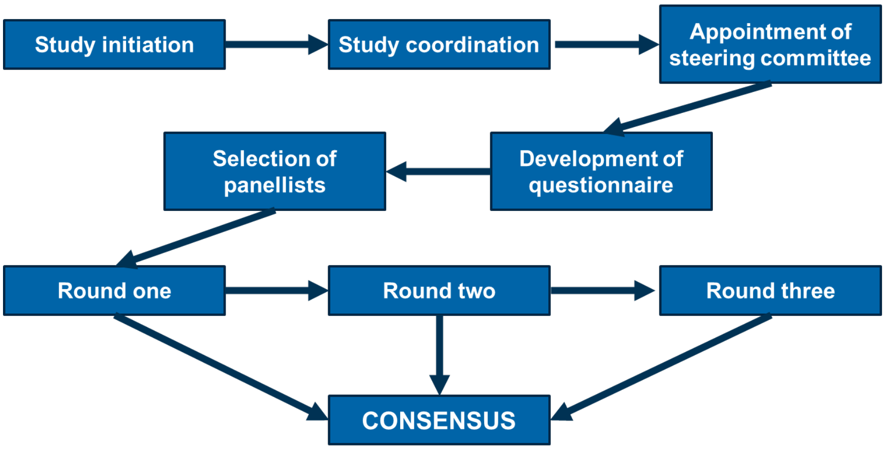

Supplementary Table 1. Characteristics of experts.

Characteristic	N (%)
Location of current clinical practice	
University hospital	17 (70.8%)
Regional hospital	6 (25%)
Other (senior consultant medical oncologist both at a regional and university hospital)	1 (4.2%)
Number of clinical experience years treating breast cancer	
6 – 10	11 (25.0%)
11 – 20	7 (29.2%)
> 20	6 (45.8%)
Highest level of experience of clinical trials participation	
National coordinating investigator	15 (62.5%)
Principal investigator	7 (29.2%)
Co-investigator	2 (8.3%)
Number of international scientific conferences with breast cancer-related topics included usually attended per year	
1	4 (16.7%)
2 – 5	20 (83.3%)

Number of breast cancer-related publications co-authored	
None	1 (4.2%)
1 – 10	7 (29.2%)
11 – 50	8 (33.3%)
>50	8 (33.3%)

Supplementary Table 2. General clinical themes.

Theme	Questions	Consensus	Consensus statement
Later treatment lines	Reason for not offering later (>3) treatment lines to mTNBC patients	100%	Impaired performance status
		62.5%	Patient willingness
DPD	DPD testing before capecitabine treatment (Q48)	100%	Yes, all patients are tested for DPD before treatment
De novo aBC disease	Statement to consider primary tumour removal	91.7%	To reduce symptoms related to primary tumour
Newly diagnosed brain metastases	Treatment choice in patients with symptomatic disease	95.8%	Radiosurgery/radiotherapy
		70.8%	Surgery
		62.5%	Systemic oncological therapy
	Treatment choice in patients with asymptomatic disease	83.3%	Radiosurgery/radiotherapy
		79.2%	Systemic oncological therapy
	Keeping current systemic treatment if extracranial disease is stable	95.8%	Yes, keep current systemic treatment

Abbreviations: aBC, advanced breast cancer; DPD, dihydropyrimidine dehydrogenase; mTNBC, metastatic triple negative breast cancer.

Supplementary Table 3. Clinical practice and local reimbursement

Theme	Questions	Consensus	Consensus statement
1L therapy	Preferred treatment choice in case of PD-L1 positive and recurrence after three years	66.7%	Atezolizumab + chemotherapy as 1st choice
		50%	Chemotherapy only in combination as 3rd choice
	Treatment choice for backbone chemotherapy if pembrolizumab with chemotherapy is chosen – recurrence three years after end of chemotherapy	NR	–
	Treatment choice for backbone chemotherapy if atezolizumab with chemotherapy is chosen – recurrence three years after end of chemotherapy	100%	Nab-paclitaxel
	Treatment choice for chemotherapy as monotherapy in case of PD-L1 positive and recurrence after three years after end of chemotherapy	NR	–
	Treatment choice for PD-L1+ and recurrence within one year	50%	Pembrolizumab + chemotherapy as 1st choice
		50%	Chemotherapy only in combination as 3rd choice
	Treatment choice for backbone chemotherapy if pembrolizumab with chemotherapy is chosen – recurrence within one year after end of chemotherapy	62.5%	Carboplatin + gemcitabine
	Treatment choice for backbone chemotherapy if atezolizumab with chemotherapy is chosen – recurrence within one year after end of chemotherapy	66.7%	Nab-paclitaxel
	Preferred chemotherapy as monotherapy in case of PD-L1 positive and recurrence within one year after end of chemotherapy	NR	–
	Preferred treatment choice in case of PD-L1 negative and recurrence after three years after end of chemotherapy	NR	–
	Preferred treatment choice in case of PD-L1 negative and recurrence within one year after end of chemotherapy	NR	–

	Time period from the end of adjuvant pembrolizumab until disease recurrence before considering initiating 1L treatment with CPI plus chemotherapy	66.7%	More than 12 months
	Treatment choice for TNBC, PD-L1– with germline BRCA mutation	62.5%	Platinum-based chemotherapy as 1st choice
		62.5%	Taxane-based chemotherapy as 3rd choice
	Treatment choice for PARP inhibitor as monotherapy in case of TNBC, PD-L1– with germline BRCA mutation	54.2%	Talazoparib
	Treatment choice for chemotherapy in case of TNBC, PD-L1– with germline BRCA mutation	83.3%	Carboplatin-based chemotherapy
2L therapy	Treatment choice for mTNBC and no germline *BRCA* mutation	NR	–
3L therapy	Treatment choice for mTNBC and no germline *BRCA* mutation	50%	Sacituzumab govitecan as monotherapy

Abbreviations: *BRCA*, breast cancer gene; CPI, checkpoint inhibitors; mTNBC, metastatic triple negative breast cancer; NR, not reached; PARP, poly-ADP ribose polymerase; PD-L1, Programmed Cell Death Ligand 1.

Supplementary Table 4. Delphi questions with options

Questions Round 1

Background question about participants

01 Where is your current clinical practice?

- University hospital
- Regional hospital
- I do not currently work in clinical practice
- Other - please specify:

02 How many years of clinical experience do you have treating breast cancer?

- >20 years
- 11-20 years
- 6-10 years
- 2-5 years
- Less than 2 years

03 What is your highest level of experience of clinical trials participation?

- National coordinating investigator
- Principal investigator
- Co-investigator
- Little or no experience

04 Please indicate approximately how many trials you have been principal, coordinating and/or co-investigator.

- Rating scale:

o National coordinating investigator [0-10, or more]

o Principal investigator [0-10, or more]

o Co-investigator [0-10, or more]

05 How many international scientific conferences (with BC-related topics included) are you usually attending per year?

- >5
- 2-5
- 1
- I do not attend international meetings

06 How many BC related publications have you co-authored?

- None
- 1-10
- 11-50
- >50

Delphi questions

Ranking treatment choices

In the following section we will ask you to rank which treatment options you choose/would choose in different contextual scenarios.

Firstly, we will ask you to rank treatment options based on your daily clinical practice *and* according to local/national reimbursement.

Secondly, we will ask the same questions, but based on current scientific evidence *and* in a scenario where all treatments in question are reimbursed.

IMPORTANT: The following baseline information is applied to all clinical scenarios: patients with performance status of 0 to 1 according to ECOG, without co-morbidities and thus suitable to all treatments.

Treatment choices in first line for patients with ER 0%, PgR 0%, HER2-neg (TNBC) and no germline BRCAmutation.

Treatment choices in first line, if the patient had a disease recurrence three years after end of adjuvant standard taxane- and anthracycline-based chemotherapy (no previous IO in neoadjuvant/adjuvant setting, no postneoadjuvant therapy). PD-L1 analysis showed CPS of 20 and PD-L1 on immune cells 10% (according to ESMO guidelines it would be PD-L1+).

Q1.01 In this clinical situation and according to local reimbursement, please rank which treatment options you prescribe most often in first line?

- Pembrolizumab + chemotherapy
- Atezolizumab + chemotherapy
- Chemotherapy only in combination
- Chemotherapy only as monotherapy
- Other (specify in comments)

Q01.02 Based on current scientific evidence, please rank which treatment options you would prescribe most often in first line? (if all treatments would be reimbursed)

- Pembrolizumab + chemotherapy
- Atezolizumab + chemotherapy
- Chemotherapy only in combination
- Chemotherapy only as monotherapy
- Other (specify in comments)

Q01.03 When you choose pembrolizumab with chemotherapy, what is your preferred option for backbone chemotherapy?

- Carboplatin + gemcitabine
- Other platinum-based chemotherapy
- Paclitaxel
- Other; please specify
- I would not choose pembrolizumab + chemotherapy

in this patient population

Q01.04 Based on current scientific evidence, what is your preferred option for backbone chemotherapy when you choose pembrolizumab with chemotherapy (if all treatments would be reimbursed)

- Carboplatin + gemcitabine
- Nab-paclitaxel
- Paclitaxel

- Other; please specify
- I would not choose pembrolizumab + chemotherapy
in this patient population

Q01.05 When you choose atezolizumab + chemotherapy, what is your preferred option for backbone chemotherapy?
- Carboplatin + gemcitabine
- Nab-paclitaxel
- Paclitaxel
- I would not choose atezolizumab + chemotherapy in
this patient population
- Other; please specify

Q01.06 Based on current scientific evidence, what is your preferred option for backbone chemotherapy when you choose atezolizumab + chemotherapy? (if all treatments would be reimbursed)

- Carboplatin + gemcitabine
- Other platinum-based chemotherapy Nab-paclitaxel
- Paclitaxel
- I would not choose atezolizumab + chemotherapy in this patient population
- Other; please specify

Q01.07 When you choose chemotherapy as monotherapy, what is your preferred option in this clinical situation and according to local reimbursement?

- Platinum compounds Taxanes
- Anthracyclines
- Capecitabine
- Eribulin
- Other, please specify

Treatment choices in first line for patients who had a disease recurrence within one year after end of adjuvant standard taxane- and anthracycline-based chemotherapy (no previous IO in neoadjuvant/adjuvant setting). PD-L1 analysis showed CPS of 20 and PD-L1 on immune cells 10%.

Q01.08 In this clinical situation and according to local reimbursement, please rank which treatment options you prescribe most often in first line?

- Pembrolizumab + chemotherapy
- Atezolizumab + chemotherapy
- Chemotherapy only in combination
- Chemotherapy only as monotherapy
- Other (specify in comments)

Q01.09 Based on current scientific evidence, please rank which treatment options you would prescribe most often in first line? (if all treatments would be reimbursed)

- Pembrolizumab + chemotherapy
- Atezolizumab + chemotherapy
- Chemotherapy only in combination
- Chemotherapy only as monotherapy

- Other (specify in comments)

Q01.10 When you choose pembrolizumab with chemotherapy, what is your preferred option for backbone chemotherapy?

- Carboplatin + gemcitabine
- Other platinum-based chemotherapy
- Nab-paclitaxel
- Paclitaxel
- Other; please specify

Q01.11 B**ased on** **current scientific evidence,** **what is your preferred option for backbone chemotherapy when you choose pembrolizumab with chemotherapy? (if all treatments would be reimbursed)**

- Carboplatin + gemcitabine
- Other platinum-based chemotherapy
- Nab-paclitaxel
- Paclitaxel
- Other; please specify

Q01.12 When you choose atezolizumab + chemotherapy, what is your preferred option for backbone chemotherapy?

- Carboplatin + gemcitabine
- Other platinum-based chemotherapy
- Nab-paclitaxel
- Paclitaxel
- Other; please specify
- I would not choose atezolizumab + chemotherapy in this patient population

Q01.13 Based on **current scientific evidence,** **what is your preferred option for backbone chemotherapy when you choose atezolizumab + chemotherapy? (if all treatments would be reimbursed)**

- Carboplatin + gemcitabine
- Other platinum-based chemotherapy
- Nab-paclitaxel
- Paclitaxel
- Other; please specify
- I would not choose atezolizumab + chemotherapy in this patient population

Q01.14 When you choose chemotherapy as monotherapy, what is your preferred option in this clinical situation and according to local reimbursement?

- Platinum compounds Taxanes
- Anthracyclines
- Capecitabine
- Eribulin
- Other, please specify

Treatment choices in first line for patients who had a disease recurrence three years after end of adjuvant standard taxane- and anthracycline-based chemotherapy but the pathology report from metastatic lesion showed a CPS lower than 10 and PD-L1 on immune cells less than 1%. No previous IO in neoadjuvant/adjuvant setting.

Q01.15 In this clinical situation and according to local reimbursement, please rank which treatment options you prescribe most often in first line?

- Platinum-based chemotherapy
- Taxane-based chemotherapy
- Anthracycline-based chemotherapy
- Carboplatin as monotherapy
- Taxanes as monotherapy
- Anthracyclines as monotherapy
- Capecitabine as monotherapy
- Eribulin as monotherapy
- Other, please specify

Treatment choices in first line for patients who had a disease recurrence within one year after end of adjuvant standard taxane- and anthracycline-based chemotherapy but the pathology report from metastatic lesion showed a CPS lower than 10 and PD-L1 on immune cells less than 1%.

Q01.16 In this clinical situation and according to local reimbursement, please rank which treatment options you prescribe most often in first line?

- Carboplatin-based chemotherapy
- Taxane-based chemotherapy
- Anthracycline-based chemotherapy
- Carboplatin as monotherapy
- Taxanes as monotherapy
- Anthracyclines as monotherapy
- Capecitabine as monotherapy

Recurrence after pembrolizumab

Q01.17 In this clinical situation and according to local reimbursement, what would be the time period from the end of adjuvant pembrolizumab until disease recurrence before you consider initiating 1st line treatment with immunotherapy plus chemotherapy?

- 0 – 6 months
- 7 – 12 months
- >12 months
- >24 months
- Other, please specify

Q01.18 Based on current scientific evidence, what would be the time period from the end of adjuvant pembrolizumab until disease recurrence before you consider initiating 1st line treatment with immunotherapy plus chemotherapy? (if all treatments would be reimbursed)

- 0 – 6 months
- 7 – 12 months
- >12 months
- >24 months
- Other, please specify

Recurrence and treating with the same chemotherapy

Q01.19 What would be the time period from the end of neoadjuvant/adjuvant chemotherapy until disease recurrence before you consider initiating 1st line treatment with the <u>same chemotherapy?</u>

- 0 – 6 months
- 7 – 12 months
- >12 months
- >24 months
- I would not consider the same chemotherapy again
- I would consider the same chemotherapy whenever the recurrence occurs
- Other, please specify

Treatment choices in first line for patients with ER 0%, PgR 0%, HER2-neg (TNBC), PD-L1 negative and with germline BRCA-mutation.

Q01.20 In this clinical situation and according to local reimbursement, please rank which treatment options you prescribe most often in 1st line?

- Platinum-based chemotherapy
- Taxane-based chemotherapy
- PARP-inhibitor
- Other (specify in comments)

Q01.21 <u>Based on current scientific evidence,</u> please rank which treatment options you would prescribe most often in 1st line? (if all treatments would be reimbursed)

- Platinum-based chemotherapy
- Taxane-based chemotherapy
- PARP-inhibitor based therapy
- Other (specify in comments)

Q01.22 When you choose PARP-inhibitor as monotherapy, what is your preferred option in this clinical situation and according to local reimbursement?

- Olaparib
- Talazoparib
- I would not choose PARP-inhibitor as monotherapy in this patient population
- Other, please specify

Q01.23 When you choose chemotherapy what is your preferred option in this clinical situation and according to local reimbursement?

- Carboplatin-based chemotherapy

Taxane-based chemotherapy

- Other, please specify

Treatment choices in second line for patients with ER 0%, PgR 0%, HER2-neg (mTNBC) and no germline BRCAmutation.

Q01.24 In this clinical situation and according to local reimbursement, please rank which treatment options you prescribe most often in 2nd line?

- Platinum-based chemotherapy

- Capecitabine as monotherapy
- Eribulin as monotherapy
- Vinorelbine as monotherapy
- Sacituzumab govitecan as monotherapy
- Other (specify in comments)

Q01.25 Based on current scientific evidence, please rank which treatment options you would prescribe most often in 2nd line? (if all treatments would be reimbursed)

- Platinum-based chemotherapy
- Capecitabine as monotherapy
- Eribulin as monotherapy
- Vinorelbine as monotherapy
- Sacituzumab govitecan as monotherapy
- Other (specify in comments)

Treatment choices in 3rd line for patients with ER 0%, PgR 0%, HER2-neg and no germline BRCA-mutation.

Q01.26 In this clinical situation and according to local reimbursement, please rank which treatment options you prescribe most often in 3rd line?

- Carboplatin-based chemotherapy
- Capecitabine as monotherapy
- Eribulin as monotherapy
- Vinorelbine as monotherapy
- Sacituzumab govitecan as monotherapy
- Other (specify in comments)

Q01.27 Based on current scientific evidence, please rank which treatment options you would prescribe most often in 3rd line? (if all treatments would be reimbursed)

- Carboplatin-based chemotherapy
- Capecitabine as monotherapy
- Eribulin as monotherapy
- Vinorelbine as monotherapy
- Sacituzumab govitecan as monotherapy
- Other (specify in comments)

Q01.28 Please rank the following clinical aspects based on their importance to make you decide not to offer later (>3) treatment lines in a patient with mTNBC?

- Impaired performance status
- Organ function deterioration
- Signs of visceral crisis
- Patient willingness
- Other (specify in comments and rank)

Independent of treatment line

Q01.29 In 2020, the European Medicines Agency recommended testing patients for dihydropyrimidine dehydrogenase (DPD) deficiency before systemic treatment with fluoropyrimidines (FP).

Do test patients for DPD before treating with capecitabine?

- Yes, all patients
- If not all – please click here to specify

Q01.30 For *de novo* aBC disease, would you consider primary tumor removal at aBC diagnosis for at least some of your patients?

- Yes
- No

Q01.30 If yes, for which patients would this be relevant?

Q01.31 Considering treatment for newly diagnosed brain metastases in patients with <u>symptomatic disease</u> – which treatment choice would you consider? Select all that apply.

- Surgery
- Radiosurgery/radiotherapy
- Systemic oncological therapy
- Other - Please specify

Q01.32 Considering treatment for newly diagnosed brain metastases in patients with <u>asymptomatic disease</u> – which treatment choice would you consider? Select all that apply.

- Surgery
- Radiosurgery/radiotherapy
- Systemic oncological therapy
- Other - Please specify

Q01.33 For a patient with newly diagnosed brain metastases: If extracranial disease is stable would you keep current systemic treatment?

- Yes
- No

Questions Round 2

Treatment choices in first line for patients with PD-L1+, ER 0%, PgR 0%, HER2-neg (TNBC) and no germline BRCA-mutation.

Q2.01 Treatment choices in first line, if the patient had a disease recurrence three years after end of adjuvant standard taxane- and anthracycline-based chemotherapy (no previous IO in neoadjuvant/adjuvant setting, no post-neoadjuvant therapy). Treatment in clinical trial is not possible.

Based on current scientific evidence and if all treatments would be reimbursed, which treatment will you most often choose in this clinical situation?

- IO treatment + chemotherapy
- Chemotherapy only in combination
- Chemotherapy only as monotherapy
- Other, please specify

Q2.02 When chemotherapy treatment is the choice in first line for patients who had a disease recurrence three years after end of adjuvant standard taxane- and anthracycline-based chemotherapy. Treatment in clinical trial is not possible.

Which treatments will you most often choose in this clinical situation (select up to 3 treatments)?

- Anthracycline-based chemotherapy
- Platinum-based chemotherapy
- Taxane-based chemotherapy
- Anthracyclines as monotherapy
- Capecitabine as monotherapy
- Carboplatin as monotherapy
- Eribulin as monotherapy
- Taxanes as monotherapy
- Other, please specify

Q2.03 When chemotherapy treatment is the choice in first line for patients who had a disease recurrence within one year after end of adjuvant standard taxane- and anthracycline-based chemotherapy Treatment in clinical trial is not possible.

Which treatments will you most often choose in this clinical situation (select up to 3 treatments)?

- Anthracycline-based chemotherapy
- Carboplatin-based chemotherapy
- Taxane-based chemotherapy
- Anthracyclines as monotherapy
- Capecitabine as monotherapy
- Carboplatin as monotherapy
- Eribulin as monotherapy
- Taxanes as monotherapy
- Other, please specify

Recurrence after pembrolizumab

Q2.04 What would be the time period from the end of adjuvant pembrolizumab until disease recurrence before you consider initiating 1st line treatment with immunotherapy plus chemotherapy? There is currently no clinical evidence available regarding this.

- > 6 months
- > 12 months
- > 24 months
- I would not consider immunotherapy again

Q2.05 Treatment choices in first line for patients with PD-L1-neg, ER 0%, PgR 0%, HER2-neg (TNBC) and with germline BRCA-mutation.
Treatment choices in first line, if the patient had a disease recurrence three years after end of adjuvant standard taxane- and anthracycline-based chemotherapy. Treatment in clinical trial is not possible.

Based on current scientific evidence and if all treatments would be reimbursed, which treatment will you most often choose in this clinical situation?

- Chemotherapy
- PARP-inhibitors
- Other (please specify)

Q2.06 Treatment choices in first line, if the patient had a disease recurrence within one year after end of adjuvant standard taxane- and anthracycline-based chemotherapy. Treatment in clinical trial is not possible.

Based on current scientific evidence and if all treatments would be reimbursed, which treatment will you most often choose in this clinical situation?

- Chemotherapy
- PARP-inhibitors
- Other (please specify)

General questions

Q2.07 Please choose up to 3 clinical aspects below that you believe have the most importance to make you decide not to offer later (>3) treatment lines in a patient with mTNBC?

- Impaired performance status
- Signs of visceral crisis / organ function deterioration
- Short duration of response in prior treatment lines
- Lack of evidence on treatment effectiveness in later treatment lines
- Patient willingness
- Other, please specify

Q2.08 In patients with de novo mBC, which of the following statements would make you consider primary tumor removal for a patient (choose all that apply)?

- To reduce tumor burden
- To reduce symptoms related to primary tumor
- To improve outcome
- To improve outcome in patients with oligometastatic disease
- To improve outcome in patients with non-visceral metastatic disease
- To improve outcome in patients responding to systemic treatment

Q2.09 Would you consider using some concurrent combination of adjuvant capecitabine, adjuvant PARP inhibitor and adjuvant immunotherapy for any triple-negative breast cancer patients with residual disease after neoadjuvant chemotherapy?

- Yes
- No

Q2.09a If yes: To which patients, please specify (Free text field)

Q2.10 Would you consider using concurrently or sequentially?

- Concurrently
- Sequentially

Disease recurrence after adjuvant capecitabine for residual disease in early-stage triple-negative breast cancer

Q2.11 A patient is treated in neoadjuvant setting with taxane-anthracycline based chemotherapy and treated with capecitabine in post-neoadjuvant setting due to residual disease. The patient relapsed with lung and bone metastasis 3 years after end of treatment with capecitabine. Relapse is negative for PD-L1 and CPS. ECOG PS 0, no comorbidities. Treatment in clinical trial is not possible.

Based on current scientific evidence and if all treatments would be reimbursed, which treatment will you most often choose in this clinical situation (select up to 3 treatments)?

- Anthracycline-based chemotherapy
- Carboplatin-based chemotherapy
- Taxane-based chemotherapy
- Capecitabine as monotherapy
- Eribulin as monotherapy
- Sacituzumab govitecan as monotherapy
- Vinorelbine as monotherapy
- Other, please specify

Q2.12 A patient with residual disease after neoadjuvant treatment with taxane-antracycline based chemotherapy, without pembrolizumab, and treated with capecitabine in post-neoadjuvant setting for residual disease. The patient relapsed with lung and bone metastasis 3 years after end of treatment with capecitabine. The patient is young, ECOG PS 0, no comorbidities. Relapse is positive for PD-L1 and CPS. Treatment in clinical trial is not possible.

Based on current scientific evidence and if all treatments would be reimbursed, which treatment will you most often choose in this clinical situation (select up to 3 treatments)?

- Anthracycline-based chemotherapy
- Carboplatin-based chemotherapy
- Immunotherapy with chemotherapy
- Taxane-based chemotherapy
- Capecitabine as monotherapy
- Eribulin as monotherapy
- Sacituzumab govitecan as monotherapy
- Vinorelbine as monotherapy
- Other, please specify

Disease recurrence after adjuvant pembrolizumab in early-stage triple-negative breast cancer

Q2.13 A patient with residual disease after neoadjuvant treatment with taxane-anthracycline based chemotherapy and pembrolizumab and treated with pembrolizumab in post-neoadjuvant setting. The patient relapsed with lung and bone metastasis 3 years after end of treatment with pembrolizumab. Relapse is negative for PD-L1 and CPS. Treatment in clinical trial is not possible.

Based on current scientific evidence and if all treatments would be reimbursed, which treatment will you most often choose in this clinical situation (select up to 3 treatments)?

- Anthracycline-based chemotherapy
- Carboplatin-based chemotherapy
- Taxane-based chemotherapy
- Capecitabine as monotherapy
- Eribulin as monotherapy
- Sacituzumab govitecan as monotherapy
- Vinorelbine as monotherapy
- Other, please specify

Q2.14 The patient relapsed 3 years after end of treatment with pembrolizumab. Relapse is positive for PD-L1 and CPS. Treatment in clinical trial is not possible.

Based on current scientific evidence and if all treatments would be reimbursed, which treatment will you most often choose in this clinical situation (select up to 3 treatments)?

- Carboplatin-based chemotherapy
- Immunotherapy with chemotherapy
- Taxane-based chemotherapy
- Anthracycline-based chemotherapy
- Capecitabine as monotherapy
- Eribulin as monotherapy
- Sacituzumab govitecan as monotherapy
- Vinorelbine as monotherapy
- Other, please specify

PATIENT CASE 1

A 30-year-old woman was diagnosed with TNBC (LABC, cT3cN1), she underwent neoadjuvant therapy with EC-90 and taxanes, followed by surgery: (CR); ypT0, pN0 (0/3), she received radiation. After 5 years of observation, she was diagnosed with liver metastases. A biopsy of the metastatic lesion; showed TNBC, BRCA-1 mut. pos.; PD-L1 neg; Treatment in clinical trial is not possible.

Q2.15 Based on <u>current scientific evidence</u> and if all treatments would be reimbursed, which treatment will you most often choose 1L in this clinical situation?

- Chemotherapy
- PARP-inhibitors

The patient received talazoparib in 1L; PD after 3 months. Lung and liver metastases were detected after 3 months.

Q2.16 Based on <u>current scientific evidence</u> and if all treatments would be reimbursed, which treatment will you most often choose in this clinical situation (select up to 3 treatments)?

- Carboplatin-based chemotherapy
- Anthracycline as monotherapy
- Capecitabine as monotherapy
- Eribulin as monotherapy
- Sacituzumab govitecan as monotherapy
- Taxanes as monotherapy
- Vinorelbine as monotherapy
- Other, please specify

Thereafter, the patient received capecitabine monotherapy with good response. New liver metastases were discovered after nine months into treatment. The patient is re-biopsied, and the pathology reports comes back: HER2 IHC 1 (HER2-low). In this clinical situation what treatment would you choose (treatment in clinical trial is not possible)?

Q2.17 Based on <u>current scientific evidence</u> and if all treatments would be reimbursed, which treatment will you most often choose in this clinical situation (select up to 3 treatments)?

- Carboplatin-based chemotherapy
- Anthracycline as monotherapy
- Eribulin as monotherapy
- Gemcitabine as monotherapy
- Sacituzumab govitecan as monotherapy
- Taxanes as monotherapy
- Trastuzumab deruxtecan as monotherapy
- Vinorelbine as monotherapy
- I would not treat patients in this setting
- Other, please specify

Thereafter, the patient received Sacituzumab govitecan with partial response for 6 months before disease progression. treatment in clinical trial is not possible. The patient is ECOG PS 0-1.

Q2.18 Which treatments will you most often choose in this clinical situation (select up to 3 treatments)?

- Anthracycline as monotherapy
- Capecitabine as monotherapy
- Carboplatin-based chemotherapy
- Eribulin as monotherapy
- Gemcitabine as monotherapy
- PARP inhibitor as monotherapy
- Taxanes as monotherapy
- Trastuzumab deruxtecan as monotherapy
- Vinorelbine as monotherapy
- I would not treat patients in this setting
- Other, please specify

PATIENT CASE 2

A 41-year-old woman was diagnosed with TNBC classified as cT1N0. She underwent breast conserving surgery and sentinel node dissection. The tumor was classified as pT1bN0. She received adjuvant chemotherapy with EC90 x 3 followed by docetaxel x 3 and radiation therapy to the breast. No gBRCA-mutation was found. Approximately 10 months after the end of adjuvant chemotherapy, she was diagnosed with liver and lung metastases. Biopsy of metastatic lesion showed TNBC with HER2 IHC 0, PD-L1 in immune cells was 8%. The patient received atezolizumab and nab-paclitaxel as 1st line therapy. A disease progression was evident after three months.

Q2.19 Which treatments would you most commonly choose as 2L treatment in this clinical situation (select up to 3 treatments)?

- Anthracycline as monotherapy
- Capecitabine as monotherapy
- Carboplatin-based chemotherapy
- Eribulin as monotherapy
- Vinorelbine as monotherapy
- Sacituzumab govitecan as monotherapy
- Other, please specify
- I would not recommend any treatment at this clinical situation

The patient received capecitabine with stable disease at the first evaluation but disease progression after five months.

Q2.20 Which treatments would you most commonly choose as 3rd line treatment in this clinical situation (select up to 3 treatments)?

- Anthracycline as monotherapy
- Carboplatin-based chemotherapy
- Eribulin as monotherapy
- Sacituzumab govitecan as monotherapy
- Vinorelbine as monotherapy
- Other, please specify
- I would not recommend any treatment at this clinical situation

Thereafter, the patient received eribulin but she had a disease progression after two months. She is clinically fit for systemic oncological therapy.

Q2.21 Which treatments would you most commonly choose as 4th line treatment in this clinical situation (select up to 3 treatments)?

- Carboplatin-based chemotherapy
- Anthracycline as monotherapy
- Sacituzumab govitecan as monotherapy
- Vinorelbine as monotherapy
- Other, please specify
- I would not recommend any treatment at this clinical situation

PATIENT CASE 3

A 61-year-old woman was diagnosed with TNBC. Pathology: 27 mm ER 5%, PR 0%, HER2 IHC 0 tumor of ductal type, grade 3, Ki67 80%, PAM50 basal like type, lymph node negative, BRCA negative. She underwent surgery with left breast conserving surgery + sentinel node. Postoperative CT scan shows small lunge metastases originating from BC, PD-L1 positive. She has comorbidities: asthma, hypertension. The patient is ECOG PS 1. One month after surgery the patient started treatment with atezolizumab + nab-paclitaxel – she developed autoimmune-hepatitis after 6 cycles – continued monotherapy with nab-paclitaxel. One month later she had progression: in lung and 3 liver metastases, continue ECOG PS 1, normal liver function. Treatment in clinical trial is not possible.

Q2.22 Which treatments will you most often choose in this clinical situation (select up to 3 treatments)?

- Anthracycline as monotherapy

- Capecitabine as monotherapy
- Carboplatin-based chemotherapy
- Gemcitabine as monotherapy
- Sacituzumab govitecan as monotherapy
- Vinorelbine as monotherapy
- I would not treat patients in this setting
- Other, please specify

The patient received capecitabine but developed multiple brain metastasis within 6 months and she received whole brain radiation. She is clinically fit for systemic oncological therapy. Treatment in clinical trial is not possible.

Q2.23 Which treatments will you most often choose in this clinical situation (select up to 3 treatments)?

- Continue capecitabine until further progression
- Carboplatin-based chemotherapy
- Gemcitabine-based chemotherapy
- Anthracycline as monotherapy
- Sacituzumab govitecan as monotherapy
- Vinorelbine as monotherapy
- I would not treat patients in this setting
- Other, please specify

PATIENT CASE 4

A 38-year-old woman, premenopausal, was diagnosed with TNBC. Pathology 26 mm (on ultrasound of breast) ER-, PR-, HER2 IHC 0, invasive ductal carcinoma, grade 3, in right breast, lymph node positive, BRCA positive. She received neoadjuvant chemotherapy – 3 cycles epirubicin + cyclophosphamide every 3 week – 3 cycles weekly paclitaxel. After neoadjuvant chemotherapy she underwent conserving surgery in the right breast + sentinel node and she received pPR. After 16 months she experienced recurrence with bone and lever metastasis, biopsy TNBC, PD-L1 negative. She received 6 cycles of carboplatin and gemcitabine – progression in liver. Treatment in clinical trial is not possible.

Q2.24 Which treatments will you most often choose in this clinical situation (select up to 3 treatments)?

- Anthracycline-based chemotherapy
- PARP inhibitor-based therapy
- Taxane-based chemotherapy
- Eribulin as monotherapy
- Sacituzumab govitecan as monotherapy
- Vinorelbine as monotherapy
- I would not treat patients in this setting
- Other, please specify

Patient received eribulin for 4 cycles – she had progression with new lung metastasis.

Q2.25 Which treatments will you most often choose in this clinical situation (select up to 3 treatments)?

- Anthracycline-based chemotherapy
- PARP inhibitor-based therapy
- Taxane-based chemotherapy
- Sacituzumab govitecan as monotherapy
- Vinorelbine as monotherapy
- I would not treat patients in this setting
- Other, please specify

PATIENT CASE 5

A 68-year old woman was diagnosed with breast cancer. Pathology: 17 mm grade III invasive ductal carcinoma, N 1/2, ER 15%, PR 0%, HER2 IHC 2+ (but ISH-), Ki-67 35%. She underwent breast conserving surgery to right breast, sentinel node biopsy. She had comorbidities: hypercholesterolemia, type 2 diabetes (with insulin), coronary artery disease (stents 5 years earlier than date of cancer diagnosis). After surgery she received (postoperatively) 1 x docetaxel + 3 x CEF. She had symptomatic pneumonitis from docetaxel, and she did not want to continue CEFs due to fatigue. She had radiotherapy to the breast and axilla (ended 6 months after surgery). Treatment with letrozole initiated.

About four years after initial diagnosis, the patient´s weight decreased ca. 5 kg over a short period of time, at the same time she experienced pain in right upper stomach. She had a whole-body CT, it showed lytic bone metastases in Th II-IV and in sacrum. Up to 10 liver metastases, ranging from 1 to 3 cm. Biopsy from the largest liver metastasis revealed ductal carcinoma, ER 5%, PR 0%, HER2 IHC 0, Ki-67 50%, PD-L1+.

At the time of metastasis, she had a PS ECOG 2, and she required daily mild opioids, and she was able to walk slowly 1 km. Height 166 cm, weight 56 kg. Transaminases slightly elevated (grade 1), bilirubin normal.

Q2.26 Which treatments will you most often choose 1L in this clinical situation (select up to 3 treatments)?

- Capecitabine
- Vinorelbine
- CDK4/6i + fulvestrant
- Immunotherapy + ChT
- Platinum-based chemotherapy
- Anthracycline-based chemotherapy
- Taxane-based chemotherapy
- Sacituzumab govitecan as monotherapy
- Trastuzumab deruxtecan as monotherapy
- I would not treat patients in this setting
- Other, please specify

Patient preferred capecitabine as she was afraid of the side effects of i.v. chemo. After 4 cycles minor response in liver, SD in bone. After 4 more cycles, she had a ECOG PS 2, and weight increase 2 kg. Able to walk slowly 1 km.

She experienced increasing sclerosis in bone metastasis, and four new liver metastases 1-2 cm each. The original liver metastases unchanged. Transaminases still slightly elevated (grade 1), bilirubin normal. Now ready to receive also i.v. chemo.

Q2.27 Which treatments will you most often choose in 2L in this clinical situation (select up to 3 treatments)?

- Continue Capecitabine
- Anthracycline-based chemotherapy
- CDK4/6i + fulvestrant
- Immunotherapy + ChT
- Platinum-based chemotherapy
- Taxane-based chemotherapy
- Vinorelbine
- Sacituzumab govitecan as monotherapy
- Trastuzumab deruxtecan as monotherapy

- I would not treat patients in this setting
- Other, please specify

Questions Round 3

Treatment choices in first line for patients with PD-L1-neg, ER 0%, PgR 0%, HER2-IHC 0/1+/2+(ISH-) (TNBC) and with germline BRCA-mutation.

Q3.01 Treatment choices in first line, if the patient had a disease recurrence three years after end of adjuvant standard taxane- and anthracycline-based chemotherapy. Treatment in clinical trial is not possible.

Based on current scientific evidence and if all treatments would be reimbursed, which treatment would you most often choose in this clinical situation?

- Chemotherapy
- PARP-inhibitors
- Other (please specify)

Q3.02 Treatment choices in first line, if the patient had a disease recurrence within one year after end of adjuvant standard taxane- and anthracycline-based chemotherapy. Treatment in clinical trial is not possible.

Based on current scientific evidence and if all treatments would be reimbursed, which treatment would you most often choose in this clinical situation?

- Chemotherapy
- PARP-inhibitors
- Other (please specify)

Disease recurrence after adjuvant capecitabine for residual disease in early-stage triple-negative breast cancer

Q3.03 A patient is treated in neoadjuvant setting with taxane-anthracycline based chemotherapy and treated with capecitabine in post-neoadjuvant setting due to residual disease. The patient relapsed with lung and bone metastasis 3 years after end of treatment with capecitabine. Relapse is negative for PD-L1 and CPS. ECOG PS 0, no comorbidities. Treatment in clinical trial is not possible.

Based on your understanding and based on current scientific evidence and if all treatments would be reimbursed, which treatment would you most often choose in this clinical situation (select up to 3 treatments)?

- Anthracycline-based chemotherapy
- Carboplatin-based chemotherapy
- Taxane-based chemotherapy
- Capecitabine as monotherapy
- Eribulin as monotherapy
- Sacituzumab govitecan as monotherapy
- Vinorelbine as monotherapy
- Other, please specify

Disease recurrence after adjuvant pembrolizumab in early-stage triple-negative breast cancer

Q3.04 The patient relapsed with lung and bone metastases 3 years after end of treatment with pembrolizumab. Relapse biopsies are negative for PD-L1 and CPS. Treatment in clinical trial is not possible.

Based on current scientific evidence and if all treatments would be reimbursed, which treatment would you most often choose in this clinical situation (select up to 3 treatments)?

- Anthracycline-based chemotherapy
- Carboplatin-based chemotherapy
- Taxane-based chemotherapy
- Capecitabine as monotherapy
- Eribulin as monotherapy
- Sacituzumab govitecan as monotherapy
- Vinorelbine as monotherapy
- Other, please specify

PATIENT CASE 1

A 30-year-old woman was diagnosed with TNBC (LABC, cT3cN1), she underwent neoadjuvant therapy with EC-90 and taxanes, followed by surgery: (CR); ypT0, pN0 (0/3), she received radiation. After 5 years of observation, she was diagnosed with liver metastases. A biopsy of the metastatic lesion; showed TNBC, BRCA-1 mut. pos.; PD-L1 neg; Treatment in clinical trial is not possible.

Q3.05 Based on current scientific evidence and if all treatments would be reimbursed, which treatment will you most often choose in this clinical situation (select up to 3 treatments)?

- Carboplatin-based chemotherapy
- Anthracycline as monotherapy
- Capecitabine as monotherapy
- Eribulin as monotherapy
- Sacituzumab govitecan as monotherapy
- Taxanes as monotherapy
- Vinorelbine as monotherapy
- Other, please specify

PATIENT CASE 3

A 61-year-old woman was diagnosed with TNBC. Pathology: 27 mm ER 5%, PR 0%, HER2 IHC 0 tumor of ductal type, grade 3, Ki67 80%, PAM50 basal like type, lymph node negative, BRCA negative. She underwent surgery with left breast conserving surgery + sentinel node. Postoperative CT scan shows small lung metastases originating from BC, PD-L1 positive. She has comorbidities: asthma, hypertension. The patient is ECOG PS 1. One month after surgery the patient started treatment with atezolizumab + nab-paclitaxel – she developed autoimmune-hepatitis after 6 cycles – continued monotherapy with nab-paclitaxel. One month later she had progression in lung and 3 new liver metastases, continued ECOG PS 1, normal liver function. Treatment in clinical trial is not possible.

The patient received capecitabine but developed multiple brain metastasis within 6 months and she received whole brain radiation. She is clinically fit for systemic oncological therapy. Treatment in clinical trial is not possible.

Q3.06 Which treatments will you most often choose in this clinical situation (select up to 3 treatments)?

- Continue capecitabine until further progression
- Carboplatin-based chemotherapy
- Gemcitabine-based chemotherapy
- Anthracycline as monotherapy

- Sacituzumab govitecan as monotherapy
- Vinorelbine as monotherapy
- I would not treat patients in this setting
- Other, please specify

PATIENT CASE 4

A 38-year-old woman, premenopausal, was diagnosed with TNBC. Pathology 26 mm (on ultrasound of breast) ER-, PR-, HER2 IHC 0, invasive ductal carcinoma, grade 3, in right breast, lymph node positive, BRCA positive. She received neoadjuvant chemotherapy – 3 cycles epirubicin + cyclophosphamide every 3 week – 3 cycles weekly paclitaxel. After neoadjuvant chemotherapy she underwent conserving surgery in the right breast + sentinel node and she received pPR. After 16 months she experienced recurrence with bone and lever metastases, biopsy TNBC, PD-L1 negative. She received 6 cycles of carboplatin and gemcitabine – progression in liver. Treatment in clinical trial is not possible.

Q3.07 Which treatments will you most often choose in this clinical situation (select up to 3 treatments)?

- Anthracycline-based chemotherapy
- PARP inhibitor-based therapy
- Taxane-based chemotherapy
- Eribulin as monotherapy
- Sacituzumab govitecan as monotherapy
- Vinorelbine as monotherapy
- I would not treat patients in this setting
- Other, please specify

PATIENT CASE 6

55-year-old woman with TNBC that recurred 4 years after adjuvant treatment. Disease was ER 0%, PgR 0%, and HER2 1+ according to IHC of a liver biopsy. No gBRCA-mutation. The patient received first line therapy with pembrolizumab and paclitaxel, there were a disease progression after 11 months. She received capecitabine as 2L with stable disease to be the best response and disease progression after 6 months.

Q3.08: Which treatments will you most often choose 3L in this clinical situation? Patient's performance status is good for any treatment option and there is no suitable clinical trial to be included.

- Anthracycline-based chemotherapy
- Platinum-based chemotherapy
- Eribulin
- Sacituzumab govitecan
- Trastuzumab deruxtecan
- Other chemotherapy
